# Trends and off-label use of botulinum toxin type A in Chinese neurological practice from 2016 to 2023: a real-world study

**DOI:** 10.3389/fphar.2026.1876015

**Published:** 2026-08-10

**Authors:** Shanshan Guo, Hailun Jiang, Jiping Huo, Zhigang Zhao, Cao Li

**Affiliations:** Department of Pharmacy, Beijing Tiantan Hospital, Capital Medical University, Beijing, China

**Keywords:** Botulinum toxin, China, neurology, off-label, trends

## Abstract

**Background:**

Botulinum toxin type A (BoNT-A) is an effective therapeutic tool for various neurological conditions, but it is frequently prescribed off-label in clinical practice. Currently, real-world prescribing trends and off-label utilization in China remain unclear. This study aimed to describe the trends in BoNT-A use, analyze off-label prescribing patterns, and evaluate the supporting scientific evidence.

**Methods:**

In this retrospective study, prescription data were extracted to analyze patient demographics, monotherapy and co-prescribing therapy of BoNT-A, and trends in BoNT-A usage from 2016 to 2023. We categorized the prescriptions into on-label and off-label groups according to the indications approved by the National Medical Products Administration of China. Additionally, the scientific evidence supporting off-label indications was evaluated based on randomized controlled trials (RCTs).

**Results:**

Our study included 9,561 BoNT-A prescriptions from patients with a median age of 59 years. Of these, 35.82% (3,425) were off-label. Over the study period, the number of BoNT-A prescriptions and the proportion of off-label use generally increased. Most prescriptions were issued for dystonia or facial nerve disorders, accounting for 51.00% and 43.48%, respectively. BoNT-A monotherapy comprised 93.54% of prescriptions, whereas among polypharmacy regimens, antiepileptics were the most frequently prescribed comedications. The majority of off-label prescriptions were issued for extrapyramidal and movement disorders, with cervical dystonia being the most frequent specific indication. Based on RCT evidence, 89.99% of off-label prescriptions were evidence-supported, peaking within extrapyramidal and movement disorders.

**Conclusion:**

This study indicated that BoNT-A prescriptions and off-label prescribing increased in Chinese neurological practice. Although such off-label use was often evidence-supported, unsubstantiated utilization may still raise potential safety concerns, highlighting the need for continuing efforts to align regulatory approvals with evolving clinical practice.

## Introduction

Botulinum toxin (BoNT) is a lethal bacterial exotoxin produced by the obligate anaerobe *Clostridium botulinum*. BoNT is composed of a 100 kDa heavy chain and a 50 kDa light chain linked by a single disulfide bond (Ayoub, 2025). The heavy chain specifically binds to receptors on the presynaptic membrane of nerve terminals, while the light chain cleaves synaptosome-associated protein-25 (SNAP-25), thereby preventing presynaptic membrane acetylcholine release and thus causing muscle paralysis (Lim and Seet, 2010). According to its antigenicity, BoNT is divided into seven major serotypes, A through G. Among these, type A BoNT (BoNT-A) was the first to be introduced and remains the most widely used clinically, due to its high affinity for neuromuscular junctions.

BoNT-A has proven effective across both cosmesis and diverse therapeutic applications. While its utility spans eight medical specialties, it features prominently within neurological practice (Jankovic, 2017). Although evidence-based reviews substantiate the clinical efficacy of BoNT-A for standard neurological conditions (Anandan and Jankovic, 2021; Sampaio Rocha-Filho et al., 2025), knowledge of its real-world prescribing trends remains limited. Real-world data on BoNT-A use are predominantly derived from European and Latin American studies, confined to specific indications such as post-stroke spasticity, multiple sclerosis, and neurogenic incontinence (Villart, 2007; Bensmail et al., 2023; Ruffion et al., 2024; Panesso et al., 2025; Bensmail et al., 2026; Van Wijk et al., 2026). To our knowledge, data on its prescribing trends within Chinese neurological practice are lacking.

Despite its broad therapeutic potential, BoNT-A has obtained formal regulatory approval for only a limited number of indications, while the majority are off-label. Currently in the United States, approved therapeutic indications for BoNT-A in adult patients include strabismus, blepharospasm, cervical dystonia, primary axillary hyperhidrosis, spasticity, chronic migraine, chronic sialorrhea, and bladder dysfunction. Off-label use provides significant therapeutic opportunities but warrants rigorous benefit-risk evaluation. A recent outbreak of iatrogenic botulism linked to off-label bariatric procedures in Europe highlighted the serious consequences of such applications, in which several patients developed severe descending paralysis (Jain et al., 2023). To understand clinical efficacy and safety, real-world prescribing data are essential. However, the available real-world evidence evaluating off-label BoNT-A use remains limited, primarily from European and Latin American studies (Villart, 2007; Matthes et al., 2010; Danes et al., 2014; Panesso et al., 2025).

In comparison, only two commercial BoNT-A formulations are approved for therapeutic use in adults in China: onabotulinumtoxinA (OnaBoNT-A, Botox®; Allergan, Irvine, CA, United States) and lanbotulinumtoxinA (LanBoNT-A, Lantox®; Lanzhou Institute of Biological Products, Lanzhou, China). Off-label prescribing patterns in China are lacking, and the extent to which these uses are supported by scientific evidence remains unclear, thereby impeding effective pharmacovigilance and regulatory decisions. To address these gaps, this real-world study was designed to (1) describe trends in BoNT-A use in Chinese neurological practice from 2016 to 2023, (2) analyze off-label prescribing patterns, and (3) evaluate the supporting scientific evidence.

## Methods

### Study design and data source

This study was a retrospective, cross-sectional analysis of BoNT-A use in the neurology department based on real-world prescription data. The data were extracted from the Hospital Prescription Analysis Cooperative Project database, which is widely utilized in pharmacoepidemiology studies (Li et al., 2022; Wu et al., 2024). This database includes prescription records from ten workdays per quarter, selected through systematic random sampling. Specifically, eligible workdays are sampled at fixed intervals to provide even temporal coverage throughout each quarter. The collected information included patient sex, age, year, region, department visited, insurance type, hospital level, source, diagnosis, medications, and drug expense. The original diagnoses were coded and classified according to the International Classification of Diseases, 10th Edition (ICD-10). The medications were similarly processed using the World Health Organization Anatomical Therapeutic Chemical (ATC) classification system. This study was approved by the Ethics Committee of Beijing Tiantan Hospital, Capital Medical University.

### Study sample

This study included BoNT-A prescriptions (ATC code: M03AX01) from the department of neurology between 2016 and 2023. Prescription information was acquired from 49 hospitals across nine cities or provinces in China, including the first-tier cities of Guangzhou, Shanghai, and Beijing, as well as the second-tier cities of Hangzhou, Shenyang, Chengdu, Tianjin, Zhengzhou, and Harbin. The extracted prescriptions contained only LanBoNT-A and OnaBoNT-A as the type of toxin. The inclusion criteria were as follows: (1) prescriptions issued between 1 January 2016 and 31 December 2023 from continuously participating hospitals; (2) the prescribed drug contained at least one BoNT-A; (3) patients were aged 18 years or older; and (4) treatment was administered within the department of neurology. Exclusion criteria: (1) duplicate prescriptions, and (2) prescriptions lacking information on patient sex, age, or diagnosis.

### Definition of off-label prescriptions

In the present study, the ICD-10 diagnosis code served as the primary treatment indication, given that 92.71% of prescriptions listed only a single diagnosis. A prescription was classified as off-label if none of the recorded diagnoses corresponded to an indication approved by the National Medical Products Administration (NMPA) as of December 2023. The specific ICD-10 codes used to define on-label indications are listed in Supplementary Table S1. For example, Meige syndrome was classified as an off-label indication because it is characterized by blepharospasm and oromandibular dystonia (Pandey and Sharma, 2017), and the latter is not an NMPA-approved indication. Similarly, focal dystonia and unspecified dystonia were classified as off-label because these diagnostic terms were insufficiently specific. This analysis focused on the assessment of off-label indications. Off-label dosages and routes of administration were not evaluated due to a lack of information regarding site-specific BoNT-A doses and precise injection techniques (e.g., intramuscular or intradermal administration).

### Scientific evidence for off-label indications

Off-label prescriptions were further classified based on the presence of scientific evidence. Using the same criteria as in our previous study (Yan et al., 2023), the term “evidence-supported” was defined as the presence of at least one randomized controlled trial (RCT) supporting the corresponding BoNT-A product for the indication, while “unsupported” denoted the absence of such evidence. As the literature on LanBoNT-A is predominantly published in Chinese, a comprehensive search was conducted both in Chinese (CNKI) and English (PubMed, the Cochrane Library) databases.

### Statistical analysis

Descriptive characteristics were performed for BoNT-A prescriptions. Overall utilization trends were quantified by the number of prescriptions, and prescribing patterns were analyzed as monotherapy or co-prescribing therapy. Continuous variables were described using median and interquartile range (IQR) owing to non-normal distribution, while categorical variables were expressed as numbers with percentages. Prescriptions were classified as on-label or off-label, with the latter further categorized into evidence-supported and unsupported groups. The Wilcoxon rank-sum test and Chi-square test were performed using SPSS (V.26). A *P* value < 0.05 was considered statistically significant.

## Results

### Descriptive statistics of total prescriptions

**FIGURE 1:**
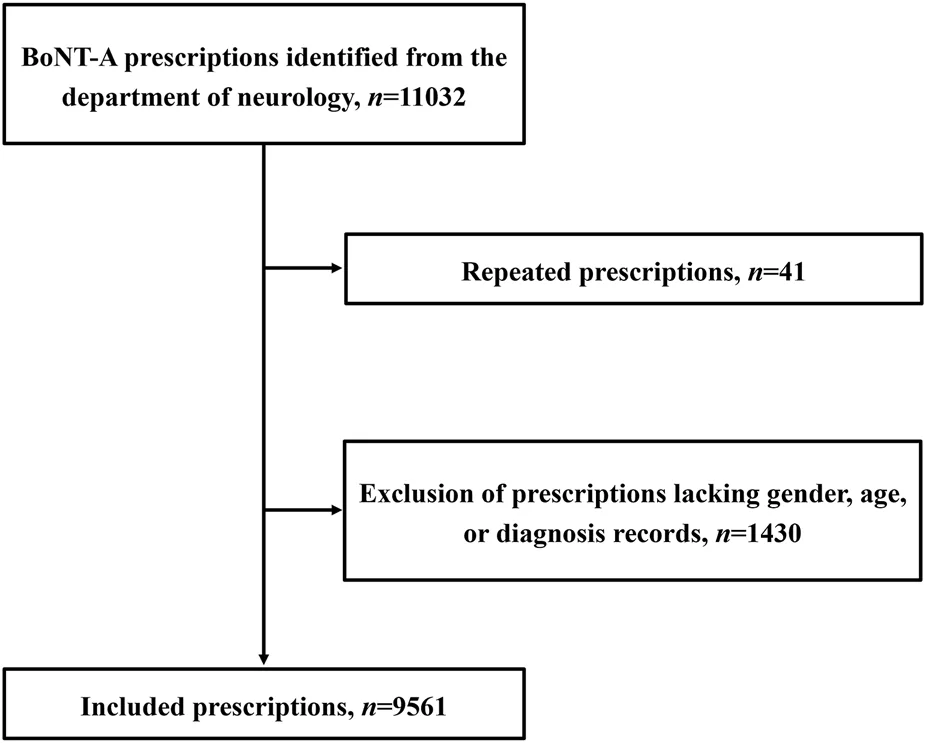
Flowchart of the prescription screening process.

**FIGURE 2:**
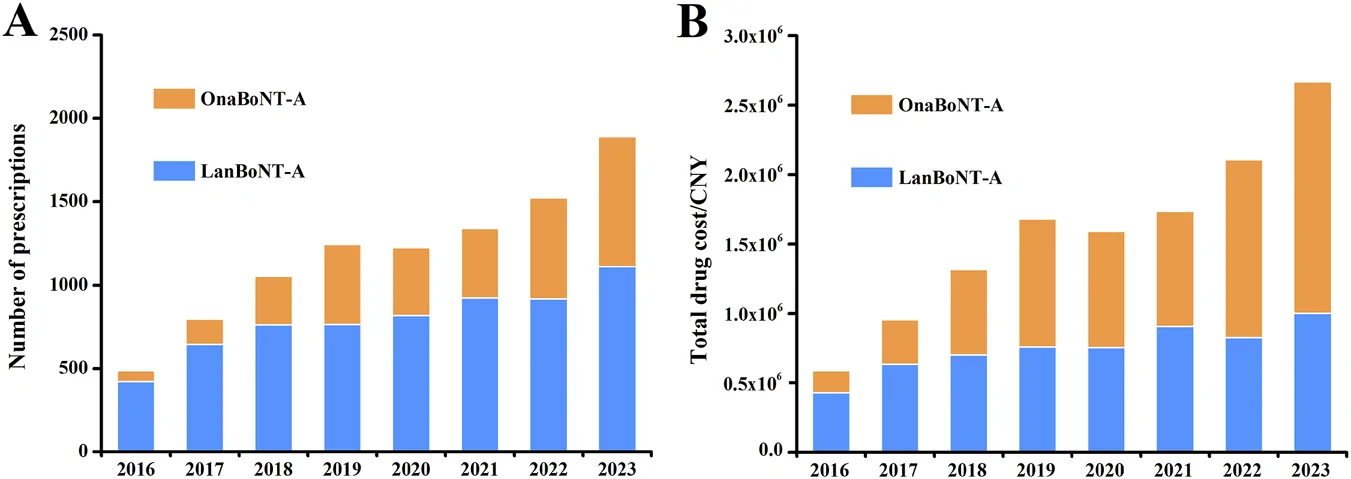
Trends in the annual number of prescriptions **(A)** and total drug cost **(B)** for BoNT-A from 2016 to 2023 (in Chinese Yuan, CNY).

A total of 9,561 prescriptions for BoNT-A were extracted from the department of neurology after applying the inclusion and exclusion criteria, comprising 6,361 for LanBoNT-A and 3,200 for OnaBoNT-A (Figure 1). Among the included prescriptions, the median age of the corresponding patients was 59 years. Of these, 66.06% were female, approximately twice the proportion of males (33.94%). The majority of prescriptions (69.52%) originated from second-tier cities and were predominantly issued in outpatient settings (97.31%) (Table 1). Furthermore, 60.83% of patients using BoNT-A had full or partial insurance coverage. Within the study database, the annual number of prescriptions for BoNT-A increased from 486 in 2016 to 1,891 in 2023, marking a 3.89-fold increase (Figure 2A; Table 1). During this period, prescriptions for OnaBoNT-A rose dramatically by approximately 12-fold, whereas those for LanBoNT-A increased only 2.64-fold. Similarly, the total annual drug cost of these two BoNT-A preparations showed a comparable upward trend, with expenditures increasing by 10.57-fold and 2.34-fold, respectively (Figure 2B). Of note, the annual expenditure for OnaBoNT-A surpassed that of LanBoNT-A starting in 2022, reflecting its increased prescription volume and higher costs.

**TABLE 1:** Demographic characteristics of patients associated with the included BoNT-A prescriptions.

Characteristic	LanBoNT-A	OnaBoNT-A	Total BoNT-A
Sex, *n* (%)			
Female	4191 (65.89)	2125 (66.41)	6316 (66.06)
Male	2170 (34.11)	1075 (33.59)	3245 (33.94)
Age, median (IQR)	60 (51–68)	58 (49–66)	59 (51–67)
Age group, *n* (%)			
18–64	4064 (63.89)	2259 (70.59)	6323 (66.13)
≥65	2297 (36.11)	941 (29.41)	3238 (33.87)
City type, *n* (%)			
First-tier	2119 (33.31)	795 (24.84)	2914 (30.48)
Second-tier	4242 (66.69)	2405 (75.16)	6647 (69.52)
Hospital level, *n* (%)			
Tertiary	6289 (98.87)	3192 (99.75)	9481 (99.16)
Secondary	72 (1.13)	8 (0.25)	80 (0.84)
Patient source, *n* (%)			
Outpatient	6142 (96.56)	3162 (98.81)	9304 (97.31)
Inpatient	219 (3.44)	38 (1.19)	257 (2.69)
Insurance type, *n* (%)			
Full or partial	3930 (61.78)	1886 (58.94)	5816 (60.83)
Self-pay	2180 (34.27)	1245 (38.91)	3425 (35.82)
N/A	251 (3.95)	69 (2.16)	320 (3.35)
Year, *n* (%)			
2016	421 (6.62)	65 (2.03)	486 (5.08)
2017	646 (10.16)	150 (4.69)	796 (8.33)
2018	761 (11.96)	293 (9.16)	1054 (11.02)
2019	764 (12.01)	480 (15.00)	1244 (13.01)
2020	819 (12.88)	406 (12.69)	1225 (12.81)
2021	922 (14.49)	419 (13.09)	1341 (14.03)
2022	917 (14.42)	607 (18.97)	1524 (15.94)
2023	1111 (17.47)	780 (24.38)	1891 (19.78)

IQR, interquartile range (P25–P75); N/A, not applicable.

The most common diagnostic category was dystonia (G24), accounting for 51.00% of all prescriptions (Table 2; Supplementary Table S2). Within this category, the three most frequent specific diagnoses were blepharospasm (G24.5, 20.94%), cervical dystonia (G24.3, 10.37%), and Meige syndrome (G24.4, 9.80%). The second most prevalent diagnostic category was facial nerve disorders (G51, 43.48%), which was overwhelmingly dominated by hemifacial spasm (G51.3, 43.30%). Additionally, a smaller proportion of prescriptions (2.44%) was associated with other neurological disorders, specifically episodic and paroxysmal disorders (G40–G47).

**TABLE 2:** Classification of diagnoses based on ICD-10 among neurological patients treated with BoNT-A, *n* (%).

ICD-10	ICD-10 grouping	LanBoNT-A (*n* = 6,361)	OnaBoNT-A (*n* = 3,200)	Total BoNT-A (*n* = 9,561)	Common diagnoses
G24	Dystonia	3127 (49.16)^a^	1749 (54.66)	4876 (51.00)	Blepharospasm, cervical dystonia, Meige syndrome
G51	Facial nerve disorders	2895 (45.51)	1262 (39.44)	4157 (43.48)	Hemifacial spasm, Bell palsy, facial myokymia
G40–G47	Episodic and paroxysmal disorders	109 (1.71)	124 (3.88)	233 (2.44)	Sleep disorder, chronic migraine, migraine
G20	Parkinson disease	62 (0.97)	30 (0.94)	92 (0.96)	Parkinson disease
G25	Other extrapyramidal and movement disorders	17 (0.27)	18 (0.56)	35 (0.37)	Facial tic, extrapyramidal syndrome, essential tremor
G50	Disorders of trigeminal nerve	21 (0.33)	1 (0.03)	22 (0.23)	Trigeminal neuralgia
F40–F48	Neurotic, stress-related and somatoform disorders	62 (0.97)	81 (2.53)	143 (1.50)	Anxiety disorder, neurotic disorder, writer’s cramp
H00–H06	Disorders of eyelid, lacrimal system and orbit	88 (1.38)	29 (0.91)	117 (1.22)	Eyelid disorder, dry eye syndrome
I60–I69	Cerebrovascular diseases	67 (1.05)	25 (0.78)	92 (0.96)	Cerebral infarction, cerebrovascular disease, sequelae of cerebral infarction
R50–R69	General symptoms and signs	40 (0.63)	46 (1.44)	86 (0.90)	Headache, hyperhidrosis, malaise and fatigue
I10–I15	Hypertensive diseases	60 (0.94)	17 (0.53)	77 (0.81)	Hypertension
R25–R29	Symptoms and signs involving the nervous and musculoskeletal systems	36 (0.57)	32 (1.00)	68 (0.71)	Tremor, spasticity, ataxia
M40–M54	Dorsopathies	31 (0.49)	15 (0.47)	46 (0.48)	Torticollis, cervical spondylosis, low back pain
R40–R46	Symptoms and signs involving cognition, perception, emotional state and behaviour	27 (0.42)	16 (0.50)	43 (0.45)	Dizziness, coma
M60–M79	Soft tissue disorders	32 (0.50)	10 (0.31)	42 (0.44)	Neuralgia, muscle hypertrophy, myositis
M00–M25	Arthropathies	15 (0.24)	7 (0.22)	22 (0.23)	Pain in joint, arthritis, arthrosis
F90–F98	Behavioural and emotional disorders with onset usually occurring in childhood and adolescence	7 (0.11)	14 (0.44)	21 (0.22)	Tic disorder
H15–H22	Disorders of sclera, cornea, iris and ciliary body	14 (0.22)	5 (0.16)	19 (0.20)	Keratoconjunctivitis sicca, keratitis
F30–F39	Mood [affective] disorders	9 (0.14)	6 (0.19)	15 (0.16)	Depressive episode
N/A	Others	339 (5.33)	139 (4.34)	478 (5.00)	Type 2 diabetes mellitus, disturbances of salivary secretion, hereditary ataxia

^a^ The proportions in parentheses represent the corresponding percentages of the column total (*n*).

### Trends in prescribing patterns

BoNT-A monotherapy was the predominant prescribing pattern, accounting for 93.54% of all prescriptions. Polypharmacy involving BoNT-A accounted for the remaining 6.46%, with proportions of two-drug (3.68%), three-drug (1.29%), and four-or-more-drug (1.50%) regimens (Table 3). Table 4 presents the detailed types of concomitant medications used in BoNT-A polypharmacy and their respective proportions among all polypharmacy prescriptions. The most frequently prescribed comedications were antiepileptics (N03, 38.51%), psychoanaleptics (N06, 26.38%), and psycholeptics (N05, 23.30%). Among two-drug polypharmacy regimens, the most common combinations were BoNT-A + clonazepam (16.99%), BoNT-A + antidepressants (5.02%), and BoNT-A + hypnotics and sedatives (4.53%) (Supplementary Table S3). For triple-drug therapy, the most frequently utilized was BoNT-A + baclofen + clonazepam (*n* = 14, 2.27%), followed by BoNT-A + clonazepam + trihexyphenidyl (*n* = 9, 1.46%), and BoNT-A + baclofen + trihexyphenidyl (*n* = 8, 1.29%).

**TABLE 3:** Trends in BoNT-A prescribing patterns in the department of neurology (2016–2023).

Prescribing pattern	Number of prescriptions (%)								
​	2016	2017	2018	2019	2020	2021	2022	2023	Total
BoNT-A monotherapy	456 (93.83)	755 (94.85)	999 (94.78)	1179 (94.77)	1151 (93.96)	1274 (95.00)	1384 (90.81)	1745 (92.28)	8943 (93.54)
Polypharmacy	30 (6.17)	41 (5.15)	55 (5.22)	65 (5.23)	74 (6.04)	67 (5.00)	140 (9.19)	146 (7.72)	618 (6.46)
2	14 (2.88)	17 (2.14)	31 (2.94)	39 (3.14)	49 (4.00)	41 (3.06)	81 (5.31)	80 (4.23)	352 (3.68)
3	6 (1.23)	8 (1.01)	9 (0.85)	16 (1.29)	10 (0.82)	10 (0.75)	32 (2.10)	32 (1.69)	123 (1.29)
≥4	10 (2.06)	16 (2.01)	15 (1.42)	10 (0.80)	15 (1.22)	16 (1.19)	27 (1.77)	34 (1.80)	143 (1.50)

**TABLE 4:** Co-medications in neurological patients treated with BoNT-A, *n* (%).

ATC	Names of medications	LanBoNT-A	OnaBoNT-A	Total BoNT-A
N03	Antiepileptics	148 (37.19)	90 (40.91)	238 (38.51)
N03AE01	Clonazepam	117 (29.40)	80 (36.36)	197 (31.88)
N03A	Other antiepileptics^a^	31 (7.79)	10 (4.55)	41 (6.63)
N06	Psychoanaleptics	107 (26.88)	56 (25.45)	163 (26.38)
N06A	Antidepressants	49 (12.31)	39 (17.73)	88 (14.24)
N06B	Psychostimulants, agents used for ADHD, and nootropics	37 (9.30)	5 (2.27)	42 (6.80)
N06D	Anti-dementia drugs	13 (3.27)	4 (1.82)	17 (2.75)
N06C	Psycholeptics and psychoanaleptics in combination	8 (2.01)	8 (3.64)	16 (2.59)
N05	Psycholeptics	83 (20.85)	61 (27.73)	144 (23.30)
N05B	Anxiolytics	29 (7.29)	32 (14.55)	61 (9.87)
N05C	Hypnotics and sedatives	28 (7.04)	21 (9.55)	49 (7.93)
N05A	Antipsychotics	26 (6.53)	8 (3.64)	34 (5.50)
N04	Anti-Parkinson drugs	87 (21.86)	50 (22.73)	137 (22.17)
N04AA01	Trihexyphenidyl	41 (10.30)	33 (15.00)	74 (11.97)
N04B	Dopaminergic agents	46 (11.56)	17 (7.73)	63 (10.19)
M03	Muscle relaxants	68 (17.09)	43 (19.55)	111 (17.96)
M03BX01	Baclofen	46 (11.56)	31 (14.09)	77 (12.46)
M03B	Other centrally acting muscle relaxants^b^	22 (5.53)	12 (5.45)	34 (5.50)
B03	Antianemic preparations	37 (9.30)	11 (5.00)	48 (7.77)
S01	Ophthalmologicals	36 (9.05)	7 (3.18)	43 (6.96)
B01	Antithrombotic agents	25 (6.28)	12 (5.45)	37 (5.99)
N02	Analgesics	21 (5.28)	16 (7.27)	37 (5.99)
C10	Lipid modifying agents	21 (5.28)	14 (6.36)	35 (5.66)
C08	Calcium channel blockers	20 (5.03)	11 (5.00)	31 (5.02)
A11	Vitamins	26 (6.53)	4 (1.82)	30 (4.85)
C09	Agents acting on the renin-angiotensin system	23 (5.78)	6 (2.73)	29 (4.69)
C07	Beta blocking agents	16 (4.02)	10 (4.55)	26 (4.21)
N/A	Others	156 (39.20)	53 (24.09)	209 (33.82)
N/A	Any polypharmacy	398 (100.00)	220 (100.00)	618 (100.00)

^a^ Other antiepileptics include oxcarbazepine (N03AF02), carbamazepine (N03AF01), topiramate (N03AX11), levetiracetam (N03AX14), and valproic acid (N03AG01).

^b^ Other centrally acting muscle relaxants include tizanidine (M03BX02) and eperisone (M03BX09).

### Analysis of off-label BoNT-A prescriptions

**FIGURE 3:**
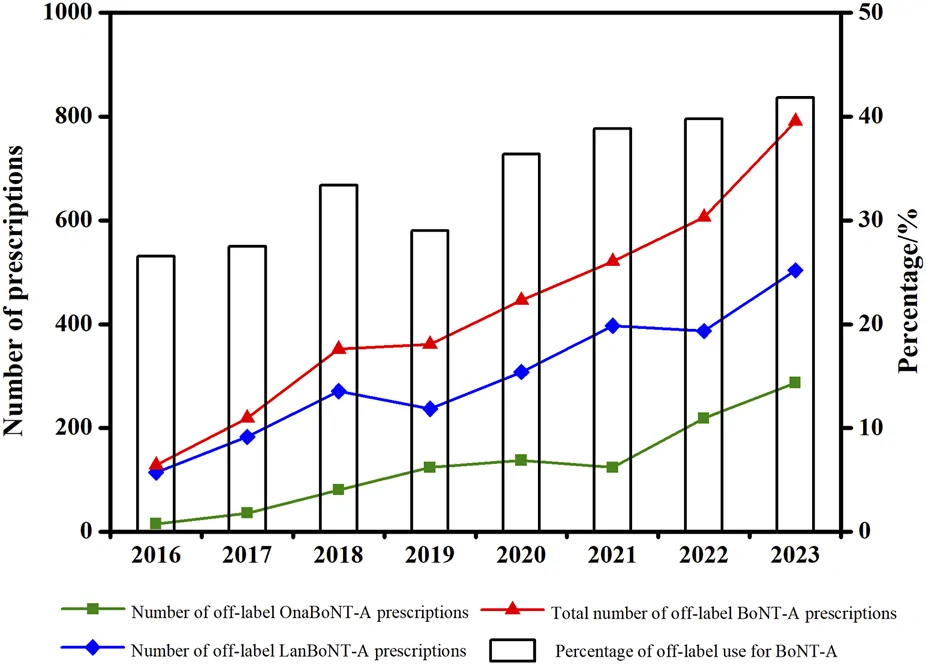
Trends in the number and proportion of off-label BoNT-A prescriptions.

Prescriptions of BoNT-A were classified as on-label or off-label based on whether at least one treatment indication was approved. Overall, 3,425 prescriptions (35.82%) were identified as off-label. Comparing the baseline characteristics of the two groups, the median age was 60 years in the on-label group and 57 years in the off-label group (Table 5). In the off-label group, prescriptions were more frequently issued to patients aged 18–64 years than to those aged ≥65 years (25.71% vs. 10.11%), with a numerical female predominance (23.46% vs. 12.36%). Significant differences were observed between the two groups in terms of formulations, age, city type, hospital level, patient source, year, and the number of diagnoses (all *P* < 0.001), as well as insurance type (*P* < 0.05). However, no statistically significant difference was found in sex (*P* = 0.378), despite an observed female predominance. The number of off-label prescriptions for BoNT-A surged from 129 in 2016 to 791 in 2023 (Figure 3). In contrast to this sharp rise in prescription counts, the proportion of off-label use increased modestly from 26.54% in 2016 to 41.83% in 2023. During the study period, the off-label rate for LanBoNT-A remained consistently higher than that for OnaBoNT-A (Supplementary Figure S1A), possibly due to its cost advantage.

**TABLE 5:** Comparison of characteristics between on-label and off-label prescriptions.

Characteristic	On-label	Off-label	*P* value
Total number, *n* (%)^a^	6136 (64.18)	3425 (35.82)	​
Formulations, *n* (%)	​	​	<0.001
LanBoNT-A	3960 (41.42)	2401 (25.11)	​
OnaBoNT-A	2176 (22.76)	1024 (10.71)	​
Sex, *n* (%)	​	​	0.378
Female	4073 (42.60)	2243 (23.46)	​
Male	2063 (21.58)	1182 (12.36)	​
Age, median (IQR)	60 (52–68)	57 (46–66)	<0.001
Age group, *n* (%)	​	​	<0.001
18–64	3865 (40.42)	2458 (25.71)	​
≥65	2271 (23.75)	967 (10.11)	​
City type, *n* (%)	​	​	<0.001
First-tier	1689 (17.67)	1225 (12.81)	​
Second-tier	4447 (46.51)	2200 (23.01)	​
Hospital level, *n* (%)	​	​	<0.001
Tertiary	6122 (64.03)	3359 (35.13)	​
Secondary	14 (0.15)	66 (0.69)	​
Patient source, *n* (%)	​	​	<0.001
Outpatient	6076 (63.55)	3228 (33.76)	​
Inpatient	60 (0.63)	197 (2.06)	​
Insurance type, *n* (%)	​	​	<0.05
Full or partial	3755 (39.27)	2061 (21.56)	​
Self-pay	2159 (22.58)	1266 (13.24)	​
N/A	222 (2.32)	98 (1.02)	​
Year, *n* (%)	​	​	<0.001
2016–2019	2519 (26.35)	1061 (11.10)	​
2020–2023	3617 (37.83)	2364 (24.73)	​
Number of diagnoses, *n* (%)	​	​	<0.001
1	5793 (60.59)	3071 (32.12)	​
2	255 (2.67)	244 (2.55)	​
≥3	88 (0.92)	110 (1.15)	​

^a^ All percentages were calculated using the total number of BoNT-A prescriptions (*n* = 9,561) as the denominator.

With respect to off-label indications, extrapyramidal and movement disorders (G20–G26) accounted for the largest number of off-label prescriptions (*n* = 3,002), followed by episodic and paroxysmal disorders (G40–G47, *n* = 233), as well as symptoms, signs and findings not elsewhere classified (R00–R99, *n* = 232) (Table 6). Among all off-label indications, cervical dystonia (G24.3) was the most commonly prescribed (*n* = 991), all of which fell within extrapyramidal and movement disorders (Table 6; Supplementary Table S2). Additionally, LanBoNT-A exhibited a higher off-label rate than OnaBoNT-A for extrapyramidal and movement disorders (66.47% vs. 48.44%), a disparity likely attributable to differences in their approved indications (Supplementary Figure S2A).

**TABLE 6:** BoNT-A prescribed for off-label indications and levels of evidence, by ICD-10.

ICD-10 grouping	ICD-10	Number of prescriptions		Level of evidence	Common off-label diagnoses
		Overall (%)^a^	Off-label (%)^b^	Evidence-supported (%)^c^	
Extrapyramidal and movement disorders	G20–G26	5004 (52.34)	3002 (59.99)	2974 (99.07)	Cervical dystonia, Meige syndrome, unspecified dystonia
Nerve, nerve root and plexus disorders	G50–G59	4181 (43.73)	41 (0.98)	22 (53.66)	Trigeminal neuralgia, Bell palsy, facial myokymia
Episodic and paroxysmal disorders	G40–G47	233 (2.44)	233 (100.00)	92 (39.48)	Sleep disorder, chronic migraine, migraine
Other diseases of the nervous system	Other G00–G99^d^	71 (0.74)	68 (95.77)	9 (13.24)	Hereditary ataxia, peripheral neuropathy, hemiplegia
Symptoms, signs and findings, not elsewhere classified	R00–R99	232 (2.43)	232 (100.00)	126 (54.31)	Headache, dizziness, tremor
Mental and behavioural disorders	F00–F99	194 (2.03)	194 (100.00)	37 (19.07)	Anxiety disorder, tic disorder, depressive episode
Diseases of the circulatory system	I00–I99	189 (1.98)	185 (97.88)	1 (0.54)	Hypertension, cerebral infarction, cerebrovascular disease
Diseases of the eye and adnexa	H00–H59	161 (1.68)	161 (100.00)	31 (19.25)	Eyelid disorder, keratoconjunctivitis sicca, dry eye syndrome
Diseases of the musculoskeletal system and connective tissue	M00–M99	120 (1.26)	120 (100.00)	82 (68.33)	Torticollis, neuralgia, muscle hypertrophy
Other diseases	N/A	299 (3.13)	299 (100.00)	49 (16.39)	Type 2 diabetes mellitus, disturbances of salivary secretion, general medical examination

^a^ Percentages were calculated using the total number of BoNT-A prescriptions (*n* = 9,561) as the denominator.

^b^ Percentages were calculated using the respective total number of prescriptions for each indication as the denominator.

^c^ Percentages were calculated using the respective total number of prescriptions for each off-label indication as the denominator.

^d^ Includes G04, G11, G12, G30, G31, G36, G37, G61, G62, G70, G72, G80, G81, G82, G90, and G93.

### Evidence evaluation for off-label BoNT-A use

The 3,425 off-label prescriptions were further classified into evidence-supported (*n* = 3,082, 89.99%) and unsupported (*n* = 343, 10.01%) groups according to the presence of scientific evidence. Baseline characteristics revealed that the evidence-supported group had a lower median age (56 vs. 61 years) and more concurrent diagnoses (2 or ≥3) than the unsupported group (Table 7). Significant differences were observed between the two groups for most variables examined, with the exception of formulations, sex, and insurance type. The number of evidence-supported prescriptions for BoNT-A continued to grow from 2016 to 2023 (Supplementary Figure S3). The proportion of evidence-supported use also generally trended upward, peaking in 2021, driven by a substantially higher rate for LanBoNT-A than for OnaBoNT-A that year (Supplementary Figures S1B, S3). With respect to off-label indications, the evidence-supported rate was highest for extrapyramidal and movement disorders (G20–G26, 99.07%) and lowest for diseases of the circulatory system (I00–I99, 0.54%) (Table 6). In addition, LanBoNT-A and OnaBoNT-A exhibited their highest and most comparable evidence-supported rates for extrapyramidal and movement disorders (99.20% vs. 98.74%) (Supplementary Figure S2B).

**TABLE 7:** Characteristics of evidence-supported versus unsupported off-label prescriptions.

Characteristic	Evidence-supported	Unsupported	*P* value
Total number, *n* (%)^a^	3082 (89.99)	343 (10.01)	​
Formulations, *n* (%)	​	​	0.190
LanBoNT-A	2150 (62.77)	251 (7.33)	​
OnaBoNT-A	932 (27.21)	92 (2.69)	​
Sex, *n* (%)	​	​	0.753
Female	2021 (59.01)	222 (6.48)	​
Male	1061 (30.98)	121 (3.53)	​
Age, median (IQR)	56 (46–65)	61 (50–69)	<0.001
Age group, *n* (%)	​	​	<0.001
18–64	2247 (65.61)	211 (6.16)	​
≥65	835 (24.38)	132 (3.85)	​
City type, *n* (%)	​	​	<0.01
First-tier	1080 (31.53)	145 (4.23)	​
Second-tier	2002 (58.45)	198 (5.78)	​
Hospital level, *n* (%)	​	​	<0.001
Tertiary	3037 (88.67)	322 (9.40)	​
Secondary	45 (1.31)	21 (0.61)	​
Patient source, *n* (%)	​	​	<0.001
Outpatient	2941 (85.87)	287 (8.38)	​
Inpatient	141 (4.12)	56 (1.64)	​
Insurance type, *n* (%)	​	​	0.531
Full or partial	1859 (54.28)	202 (5.90)	​
Self-pay	1138 (33.23)	128 (3.74)	​
N/A	85 (2.48)	13 (0.38)	​
Year, *n* (%)	​	​	<0.001
2016–2019	923 (26.95)	138 (4.03)	​
2020–2023	2159 (63.04)	205 (5.99)	​
Number of diagnoses, *n* (%)	​	​	<0.001
1	2743 (80.09)	328 (9.58)	​
2	237 (6.92)	7 (0.20)	​
≥3	102 (2.98)	8 (0.23)	​

^a^ All percentages were calculated using the total number of off-label BoNT-A prescriptions (*n* = 3,425) as the denominator.

## Discussion

In this retrospective study, we analyzed BoNT-A prescribing patterns, off-label use, and the supporting scientific evidence in neurological practice from 2016 to 2023. To the best of our knowledge, this is the first study to describe off-label BoNT-A use in Chinese neurological practice. It should be noted that our study included only two BoNT-A formulations—LanBoNT-A and OnaBoNT-A. The other two BoNT-A preparations approved in China during the study period, abobotulinumtoxinA (Dysport®; Ipsen, Slough, UK) and letibotulinumtoxinA-wlbg (Letybo®; Hugel Pharma Inc., Seoul, Korea), were not included, as they were authorized only for cosmetic indications without medical applications. Furthermore, we noted that LanBoNT-A has not been approved by the Food and Drug Administration (FDA). Significant discrepancies exist between the indications authorized by the FDA and those approved by the NMPA for OnaBoNT-A. Consequently, off-label use in this study was defined based on NMPA-approved indications, as this approach aligns more closely with the circumstances in China. As a result, our findings may differ from those of studies conducted in other countries.

Our study demonstrated an increase in BoNT-A prescribing and off-label use between 2016 and 2023. Notably, prescriptions decreased slightly in 2020, which may be related to COVID-19 that temporarily restricted patient access to hospitals (Santamato et al., 2021). In addition, we observed an overall rise in off-label prescribing, which may reflect a gap between regulatory authorization and established clinical practice. For instance, while BoNT-A is a global standard treatment for cervical dystonia (Albanese et al., 2015), this use is not approved by the NMPA. Furthermore, the off-label rate for LanBoNT-A remained consistently higher than that for OnaBoNT-A throughout the study period. Since off-label use is typically excluded from insurance coverage, the resulting financial burden may contribute to a preference for LanBoNT-A due to its lower cost (Fang et al., 2020). This aligns with evidence that more economical BoNT-A alternatives significantly reduce the burden on both payers and patients (Kazerooni and Watanabe, 2021).

BoNT-A utilization in this study was primarily observed in patients with a median age of 59 years, consistent with the late-adulthood age profile of dystonia in China. Previous epidemiological research in Zhejiang reported a mean age of 53.82 years for primary dystonia (Wang et al., 2016). Similarly, studies from Taiwan on blepharospasm identified a mean age of 58 years, with a peak incidence in the 50–59 age group (Hwang, 2012; Sun et al., 2018). Although an earlier Chinese survey recorded a younger mean age at onset for hemifacial spasm (46.6 ± 11.5 years) (Wang et al., 2014), our findings align more closely with recent international data from Finland, where the mean age at diagnosis was 56.47 years (Nurminen et al., 2023). Moreover, the overall female-to-male ratio among patients treated with BoNT-A was approximately 2:1 (66.06% vs. 33.94%), which is consistent with previous epidemiological observations. For dystonia, a large-scale international analysis reported an overall female-to-male ratio of 2.4:1 (Kilic-Berkmen et al., 2024). This trend is mirrored in China, where primary dystonia studies have reported female-to-male ratios ranging from 1.48:1 to 3:1 (Wang et al., 2012; Wang et al., 2016). Furthermore, a female-to-male ratio of 1.8:1, as previously documented in Chinese patients with hemifacial spasm (Wang et al., 2014), was consistent with the demographic profile observed in our real-world practice.

The most common diagnostic category for BoNT-A prescriptions in our study was dystonia (G24, 51.00%), primarily comprising blepharospasm (G24.5, 20.94%), cervical dystonia (G24.3, 10.37%), and Meige syndrome (G24.4, 9.80%). This high utilization is consistent with major international guidelines that endorse BoNT-A as a crucial therapeutic option for various dystonias (Albanese et al., 2011; Simpson et al., 2016). Although this distribution of dystonia subtypes differs from that reported in Europe, where cervical dystonia is the most prevalent subtype (Dressler et al., 2022), our findings align with previous epidemiological data demonstrating that blepharospasm is the most common focal dystonia in China (Wang et al., 2012; Wang et al., 2016). Furthermore, the notable proportion of prescriptions for Meige syndrome reflects the effectiveness of BoNT-A in managing this complex dystonia (Jochim et al., 2020). Of note, diagnostic ambiguity may limit the precision of this subtype distribution, as 8.32% of prescriptions listed a diagnosis of unspecified dystonia. The second most prevalent diagnostic category was facial nerve disorders (G51, 43.48%), which was predominantly driven by hemifacial spasm (G51.3, 43.30%). Previous studies indicate a higher prevalence of hemifacial spasm in Asian populations compared with Western countries (Xiang et al., 2024). Systematic reviews have confirmed the symptom relief and favorable safety profile of BoNT-A for hemifacial spasm (Duarte et al., 2020), further supporting its widespread application in neurological practice.

In the present study, BoNT-A monotherapy accounted for the vast majority (93.54%) of all prescriptions. This predominance of monotherapy is consistent with international data showing that most patients with dystonia receive BoNT-A alone (Richardson et al., 2017). In clinical practice, BoNT-A is recognized as the primary treatment for most individuals with focal or segmental dystonia (Mohamed et al., 2024). In instances of polypharmacy, BoNT-A was most frequently co-prescribed with antiepileptics (N03). This trend parallels observations in other large cohort studies, where antiepileptics are frequently reported as concomitant medications for patients receiving BoNT-A for cervical dystonia (Hull et al., 2023). However, antiepileptics might induce iatrogenic movement disorders, particularly parkinsonism and tremor (Saenz-Farret et al., 2022). Clonazepam (N03AE01) was the most frequently co-prescribed antiepileptic in our study, consistent with recent findings identifying it as the most commonly prescribed benzodiazepine for blepharospasm and Meige syndrome (Heo et al., 2025). Despite its widespread use, the clinical efficacy of clonazepam remains limited for these conditions and unsubstantiated for hemifacial spasm (Seol-Hee et al., 2022). Overall, these findings support BoNT-A monotherapy as the standard pharmacotherapeutic approach and indicate that concomitant medications should be prescribed judiciously rather than routinely.

Furthermore, we observed an off-label rate of 35.82% for BoNT-A prescriptions. Studies investigating off-label BoNT-A use remain limited, with only 3 European studies and one from Latin America. While most previous investigations were limited by short observational periods (1–3 years) or relied on questionnaire surveys, our study provides an eight-year analysis of neurological practice, offering insights into the evolution of off-label prescribing. Our rate is higher than the 7.6% reported in a recent Latin American study, primarily driven by the broader range of neurological indications approved by their local regulatory authority, including essential tremor, myoclonus, and tension-type headache (Panesso et al., 2025). Nevertheless, extensive off-label BoNT-A use remains a globally recognized phenomenon. For instance, an evaluation from a European rehabilitation hospital reported an off-label rate of 74% in 2003, prior to the expansion of approved indications (Villart, 2007), while a separate survey found that 75% of treated indications in otorhinolaryngology were off-label (Matthes et al., 2010). Additionally, a southern European multicenter study identified BoNT-A as one of the most frequently prescribed off-label medications (Danes et al., 2014). These exceptionally high rates in earlier European studies were potentially driven by the time lag between emerging clinical evidence and formal regulatory approvals. It is worth noting that unspecified dystonia (G24.9) accounted for 22.45% of off-label prescriptions, suggesting that the reported off-label rate should be interpreted cautiously, as some may represent approved indications. Meanwhile, off-label BoNT-A use may pose safety concerns, including systemic toxin spread and iatrogenic botulism (Dressler et al., 2026). Therefore, continued pharmacovigilance and careful clinical monitoring are warranted when considering off-label BoNT-A treatment.

We observed substantial off-label use of BoNT-A for extrapyramidal and movement disorders (G20–G26), which also showed the highest evidence-supported rate (99.07%). This pattern may be driven by the discrepancy between evidence supporting BoNT-A for various focal dystonias (e.g., cervical, oromandibular, and laryngeal dystonias) and its limited NMPA approval, which is largely restricted to blepharospasm (Comella, 2018; Rodrigues et al., 2020; Hyodo et al., 2021). According to our results, cervical dystonia (G24.3) was the most frequent off-label indication. This aligns with international guidelines and Chinese expert consensus statements, which recommend BoNT-A as first-line therapy for cervical dystonia (Albanese et al., 2011; Simpson et al., 2016; Expert group on the therapeutic application of botulinum toxin, 2018). Furthermore, systematic reviews suggest that BoNT-A may be more effective, better tolerated, and safer than traditional anticholinergics (Rodrigues et al., 2021). Thus, the frequent off-label use of BoNT-A for cervical dystonia reflects a regulatory gap in which formal approval may not have kept pace with established clinical evidence. The second most common off-label diagnostic category was episodic and paroxysmal disorders (G40–G47), predominantly consisting of prescriptions for sleep disorders. Specifically, these prescriptions may reflect the use of BoNT-A for sleep bruxism. Systematic reviews suggest that BoNT-A may reduce bruxism symptoms; however, larger studies with longer follow-up are needed to further validate its efficacy (Sendra et al., 2024; Yacoub et al., 2025).

The 89.99% evidence-supported rate for off-label BoNT-A prescriptions in our study exceeds previously reported rates (54.2%–65.8%) in Chinese neurological practice, suggesting a relatively cautious approach to the off-label application of BoNT-A (Yi et al., 2012). The use of BoNT-A for diseases of the circulatory system (I00–I99) was also observed, with cerebral infarction being one of the most common recorded diagnoses. These prescriptions may have represented BoNT-A treatment for post-stroke spasticity. Although BoNT-A is approved in China for post-stroke upper limb spasticity, a standalone diagnosis of cerebral infarction was deemed unsupported, given that approximately 75% of patients do not develop post-stroke spasticity (Zeng et al., 2021). Unsubstantiated BoNT-A injection may impair motor control of the injected muscles and potentially compromise residual motor function (Santamato and Panza, 2017). These results underscore the importance of careful risk-benefit assessment during clinical decision-making.

Our study had several limitations. First, our data were predominantly obtained from tertiary hospitals (99.16%), which may limit the generalizability of the findings. Accordingly, our results should be interpreted with caution when generalizing beyond tertiary hospital settings. Second, diagnostic data within the database lacked sufficient clinical specificity because diagnoses were coded using the ICD-10 system, which may not fully capture specific clinical subtypes, treatment goals, or complication status. For example, a physician may record G24.9 (unspecified dystonia) without providing a specific dystonia subtype to confirm compliance with approved indications; therefore, such cases were conservatively classified as off-label. Similarly, classification uncertainty may arise in records of cerebral infarction if post-stroke upper limb spasticity was not explicitly documented. Third, the database lacked detailed BoNT-A treatment and follow-up data, such as site-specific dosing, injection technique, effectiveness, adverse effects, and treatment continuation, thereby limiting a more detailed assessment of the prescribing patterns reported in this study. Fourth, our estimate of off-label BoNT-A prescribing was conservative because we did not assess other dimensions of off-label use (e.g., dosage and route of administration). Finally, the overall evidence-supported rate may appear higher because the classification was based on the availability of at least one RCT rather than on a comprehensive evidence-grading system.

## Conclusion

In this study, BoNT-A prescriptions and off-label prescribing increased in Chinese neurological practice from 2016 to 2023. The overall off-label rate of BoNT-A was 35.82%. The majority of off-label prescriptions were issued for extrapyramidal and movement disorders, with cervical dystonia being the most frequent specific indication. Although such off-label prescribing was often supported by scientific evidence, unsubstantiated utilization may still raise safety concerns. Continued efforts are necessary to ensure evidence-based use and to drive regulatory advancements to reflect evolving clinical practice.

## Data Availability

The original contributions presented in the study are included in the article/Supplementary Material, further inquiries can be directed to the corresponding authors.
