# Supplementary material for: Trends and off-label use of botulinum toxin type A in Chinese neurological practice from 2016 to 2023: a real-world study
Source: Front Pharmacol. 2026 Aug 10;17:1876015. doi: 10.3389/fphar.2026.1876015 (PMC13501383; doi:10.3389/fphar.2026.1876015)
Supplement: Supplementary file 1 [file Supplementaryfile1.docx]

Supplementary Material

**Supplementary Table S1** ICD-10 codes used to define on-label indications for BoNT-A in this study.

| **Formulation** | **On-label indications^a^** | **ICD-10** |
| --- | --- | --- |
| LanBoNT-A | Blepharospasm | G24.5 |
|  | Hemifacial spasm | G51.3 |
|  | Post-stroke upper limb spasticity^b^ | (I60–I64, I69.0–I69.4) and (G81.1, G82.4) |
| OnaBoNT-A | Blepharospasm | G24.5 |
|  | Hemifacial spasm | G51.3 |

^a^ Only NMPA-approved indications documented in the analyzed prescriptions are listed.

^b^ Defined as the concurrent presence of a diagnosis of stroke (or its sequelae) and either spastic hemiplegia (G81.1) or spastic tetraplegia (G82.4).

**Supplementary Table S2** Common diagnoses among neurological patients treated with BoNT-A, *n* (%).

| **ICD-10** | **Diagnoses** | **LanBoNT-A**  **(*n* = 6,361)** | **OnaBoNT-A**  **(*n* = 3,200)** | **Total BoNT-A**  **(*n* = 9,561)** |
| --- | --- | --- | --- | --- |
| G51.3 | Hemifacial spasm | 2888 (45.40)^a^ | 1252 (39.13) | 4140 (43.30) |
| G24.5 | Blepharospasm | 1075 (16.90) | 927 (28.97) | 2002 (20.94) |
| G24.3 | Cervical dystonia | 720 (11.32) | 271 (8.47) | 991 (10.37) |
| G24.4 | Meige syndrome | 698 (10.97) | 239 (7.47) | 937 (9.80) |
| G24.9 | Unspecified dystonia | 514 (8.08) | 281 (8.78) | 795 (8.32) |
| G24.8 | Focal dystonia | 120 (1.89) | 31 (0.97) | 151 (1.58) |
| G47.9 | Sleep disorder | 59 (0.93) | 63 (1.97) | 122 (1.28) |
| F41.9 | Anxiety disorder | 52 (0.82) | 64 (2.00) | 116 (1.21) |
| H02.9 | Eyelid disorder | 76 (1.19) | 27 (0.84) | 103 (1.08) |
| G20.x | Parkinson disease | 62 (0.97) | 30 (0.94) | 92 (0.96) |
| I10.x | Hypertension | 60 (0.94) | 17 (0.53) | 77 (0.81) |
| R51.x | Headache | 31 (0.49) | 34 (1.06) | 65 (0.68) |
| G44.0 | Chronic migraine | 20 (0.31) | 33 (1.03) | 53 (0.55) |
| R42.x | Dizziness | 26 (0.41) | 16 (0.50) | 42 (0.44) |
| G43.9 | Migraine | 12 (0.19) | 25 (0.78) | 37 (0.39) |
| G25.x | Other extrapyramidal and movement disorders^b^ | 17 (0.27) | 18 (0.56) | 35 (0.37) |
| I63.9 | Cerebral infarction | 23 (0.36) | 11 (0.34) | 34 (0.36) |
| R25.1 | Tremor | 16 (0.25) | 16 (0.50) | 32 (0.33) |
| R25.2 | Spasticity | 17 (0.27) | 14 (0.44) | 31 (0.32) |
| M43.6 | Torticollis | 18 (0.28) | 10 (0.31) | 28 (0.29) |
| I67.9 | Cerebrovascular disease | 19 (0.30) | 5 (0.16) | 24 (0.25) |
| E11.9 | Type 2 diabetes mellitus | 17 (0.27) | 5 (0.16) | 22 (0.23) |
| G50.0 | Trigeminal neuralgia | 21 (0.33) | 1 (0.03) | 22 (0.23) |
| F95.9 | Tic disorder | 7 (0.11) | 14 (0.44) | 21 (0.22) |
| M79.2 | Neuralgia | 15 (0.24) | 4 (0.13) | 19 (0.20) |
| H16.2 | Keratoconjunctivitis sicca | 12 (0.19) | 5 (0.16) | 17 (0.18) |
| K11.7 | Disturbances of salivary secretion | 10 (0.16) | 7 (0.22) | 17 (0.18) |
| I69.3 | Sequelae of cerebral infarction | 12 (0.19) | 4 (0.13) | 16 (0.17) |
| M62.8 | Muscle hypertrophy | 11 (0.17) | 5 (0.16) | 16 (0.17) |
| F32.9 | Depressive episode | 9 (0.14) | 6 (0.19) | 15 (0.16) |
| N/A | Others | 421 (6.62) | 191 (5.97) | 612 (6.40) |

^a^ The proportions in parentheses represent the corresponding percentages of the column total (*n*).

^b^ Other extrapyramidal and movement disorders include facial tic (G25.6), extrapyramidal syndrome (G25.9), essential tremor (G25.0), myoclonus (G25.3), and restless legs syndrome (G25.8).

**Supplementary Table S3** Trends in the top 10 dual-therapy combinations prescribed from 2016 to 2023.

|  | **Number of prescriptions (%)** | | | | | | | | |
| --- | --- | --- | --- | --- | --- | --- | --- | --- | --- |
|  | **2016** | **2017** | **2018** | **2019** | **2020** | **2021** | **2022** | **2023** | **Total** |
| BoNT-A + Clonazepam | 3 (10.00)^a^ | 3 (7.32) | 8 (14.55) | 8 (12.31) | 18 (24.32) | 12 (17.91) | 26 (18.57) | 27 (18.49) | 105 (16.99) |
| BoNT-A + Antidepressants | 0 (0) | 3 (7.32) | 0 (0) | 4 (6.15) | 4 (5.41) | 6 (8.96) | 6 (4.29) | 8 (5.48) | 31 (5.02) |
| BoNT-A + Hypnotics and sedatives | 0 (0) | 2 (4.88) | 1 (1.82) | 6 (9.23) | 2 (2.70) | 3 (4.48) | 10 (7.14) | 4 (2.74) | 28 (4.53) |
| BoNT-A + Anxiolytics | 1 (3.33) | 0 (0) | 1 (1.82) | 1 (1.54) | 5 (6.76) | 5 (7.46) | 6 (4.29) | 5 (3.42) | 24 (3.88) |
| BoNT-A + Ophthalmic hyaluronic acid | 0 (0) | 0 (0) | 2 (3.64) | 2 (3.08) | 0 (0) | 2 (2.99) | 4 (2.86) | 12 (8.22) | 22 (3.56) |
| BoNT-A + Other antiepileptics^b^ | 1 (3.33) | 4 (9.76) | 3 (5.45) | 3 (4.62) | 4 (5.41) | 0 (0) | 1 (0.71) | 2 (1.37) | 18 (2.91) |
| BoNT-A + Baclofen | 1 (3.33) | 1 (2.44) | 3 (5.45) | 2 (3.08) | 2 (2.70) | 1 (1.49) | 5 (3.57) | 0 (0) | 15 (2.43) |
| BoNT-A + Lidocaine for topical use | 1 (3.33) | 0 (0) | 2 (3.64) | 1 (1.54) | 2 (2.70) | 1 (1.49) | 5 (3.57) | 2 (1.37) | 14 (2.27) |
| BoNT-A + Trihexyphenidyl | 2 (6.67) | 1 (2.44) | 0 (0) | 1 (1.54) | 1 (1.35) | 1 (1.49) | 3 (2.14) | 4 (2.74) | 13 (2.10) |
| BoNT-A + Beta blocking agents | 0 (0) | 2 (4.88) | 1 (1.82) | 3 (4.62) | 2 (2.70) | 0 (0) | 2 (1.43) | 0 (0) | 10 (1.62) |

^a^ Percentages were calculated using the annual number of polypharmacy prescriptions as the denominator.

^b^ Other antiepileptics include oxcarbazepine (N03AF02), carbamazepine (N03AF01), topiramate (N03AX11), and valproic acid (N03AG01).

**Supplementary Figure S1** Trends in off-label (A) and evidence-supported (B) use for BoNT-A.

**Supplementary Figure S2** Proportions of off-label (A) and evidence-supported (B) use for BoNT-A, by neurological disease category (ICD-10 G00–G99).

**Supplementary Figure S3** Trends in evidence-supported off-label BoNT-A prescriptions.
